# Editorial: Harnessing physiological synchronization and hyperscanning to enhance collaboration and communication

**DOI:** 10.3389/fnrgo.2022.956087

**Published:** 2022-08-12

**Authors:** Vesna Dominika Novak, Theodoros Kostoulas, Michal Muszynski, Caterina Cinel, Anton Nijholt

**Affiliations:** ^1^Department of Electrical Engineering and Computer Science, University of Cincinnati, Cincinnati, OH, United States; ^2^Department of Information and Communication Systems Engineering, University of the Aegean, Samos, Greece; ^3^Department of Basic Neurosciences, University of Geneva, Geneva, Switzerland; ^4^BCI-NE Lab, School of Computer Science and Electronic Engineering, University of Essex, Colchester, United Kingdom; ^5^Human Media Interaction, University of Twente, Enschede, Netherlands

**Keywords:** affective computing, collaboration, communication, hyperscanning, human-computer interaction, physiological synchronization

## Introduction

Physiological synchronization is a phenomenon in which the central and autonomic nervous system responses of two or more individuals become increasingly correlated as the individuals work together or simply talk to each other (Noy et al., 2015; Ahonen et al., 2016; Vanutelli et al., 2017). This synchronization can be measured using a variety of sensors; in the area of brain signals, it is analyzed *via* hyperscanning (multi-brain measurement) techniques (Vanutelli et al., 2017; Liu et al., 2021). Furthermore, the synchronization occurs involuntarily and provides rich information about social functioning and relationships. For example, during collaborative tasks, it reflects the collaboration quality (Szymanski et al., 2017; Reinero et al., 2021) as well as role dynamics (e.g., leaders vs. followers; Konvalinka et al., 2014). In conversational scenarios, it reflects the degree of rapport, for example, between a therapist and their client (Bar-Kalifa et al., 2019; Tschacher and Meier, 2020) or a teacher and their students (Pan et al., 2020; Sun et al., 2020; Zheng et al., 2020). Finally, during shared experiences such as movie watching, it reflects moments of shared engagement (Muszynski et al., 2018).

Since physiological synchronization occurs automatically and can be measured in real-time during collaboration or communication, it could be used to enhance interpersonal interaction in diverse ways. For example, in team scenarios, physiological synchronization could be used to identify human workers who collaborate effectively with each other, and this information could then be used to assemble more efficient teams. Alternatively, synchronization could be visually presented to participants as a form of biofeedback that would allow them to change their behavior; for example, in education, it would allow teachers to better gauge which students are engaged and focus on unengaged students. Finally, the information could be used by developers to improve the design of future group activities, for example, to identify the most engaging activities in a multiplayer game and redesign the game to be more focused on such activities.

So far, most studies in the area of physiological synchronization and hyperscanning have involved offline analysis of large samples. However, researchers are increasingly using this technology to enhance real-time collaboration and communication. This Research Topic thus aims to showcase emerging research on single-trial interpretation/visualization of physiological synchronization as well as intelligent machine adaptation and audiovisual feedback based on this physiological information. It is our hope that this Research Topic will lead to more widespread adoption of physiological synchronization and hyperscanning as technological tools for enhancement of collaboration and communication, improving human health and wellbeing in countless ways.

## Contributions

This Research Topic includes five peer-reviewed original research papers with original research.

Nozawa et al. used functional near-infrared spectroscopy to evaluate interbrain synchronization in a group language learning scenario. They showed that students working in the same group exhibited significantly higher interbrain synchronization than students belonging to separate groups. Furthermore, the degree of interbrain synchronization was significantly correlated with self-reported flow dynamics. The study suggests that synchronization can therefore provide a quantitative measure of the motivational dynamics during collaborative learning. As a next step, the researchers propose providing synchronization feedback to teachers and students in order to facilitate further collaboration and identify left-behind students, potentially significantly improving learning outcomes.

Chen et al. discuss the design and the validation of “Hybrid Harmony,” an open-source software package that supports simultaneous recording of multiple electroencephalographic (EEG) data streams as well as real-time visualization and sonification of intersubject synchrony. Hybrid Harmony is meant to be used by researchers to study the effects of synchrony feedback to improve social interaction. Other potential users are biofeedback artists and multiplayer game developers. The main feature of Hybrid Harmony is that users can select and explore various synchrony metrics for different (social) contexts. From the validation, using a large dataset of dyadic interactions, the authors conclude that synchrony measures obtained with their tool correlate with socially relevant features of interactions.

Susnoschi Luca et al. developed and evaluated EEG-based neurofeedback for collaborative and competitive gaming. Participants were required to either keep their relative alpha power similar (collaboration) or exhibit higher relative alpha power than their opponent (competition). The researchers found that interbrain synchrony existed during gaming but not during baseline. In collaborating dyads, participants with higher resting relative alpha power were more active in regulating their brain activity. In competing dyads, losing players were not able to relax and thus exhibited higher engagement of frontal areas. The study demonstrates possible implementations of hyperscanning for competitive and collaborative games, and may broadly impact multiplayer gaming.

Stuldreher et al. attempted to identify individuals sharing the same attentional focus based on physiological synchrony. All participants listened to the same stimulus (an audiobook), but one group was asked to focus on the narrative and the other was asked to focus on intermittent audio and other stimuli over the course of the narrative. EEG, electrodermal activity, and electrocardiography signals were recorded and used with different unsupervised learning algorithms were used. Unimodal results only reached accuracies above the chance level for the EEG. However, combining information from multiple modalities yielded higher accuracy than using EEG alone.

Boyd et al. investigated the role of physiological linkage in social interactions. To do so, they studied electrocardiography signals obtained from dyads of strangers. The researchers determined three prototypical patterns that characterize physiological linkage, in terms of differences in frequency of oscillation, phase, damping, and amplification. They found that individual differences and affiliation could predict physiological linkage patterns. This study highlights the importance of considering interpersonal differences when investigating physiological linkage, as well as the value of capturing dynamic patterns of that linkage.

## Conclusion

Research on physiological synchronization and hyperscanning is progressing at a rapid pace, with new methods and application areas constantly appearing. In general, physiological synchronization can occur in diverse social interaction scenarios—collaboration, communication, or competition within dyads or larger groups. The degree of synchronization is visible in amplitudes and phases of physiological signals at different granularity levels and can be measured and quantified nearly in real time. Furthermore, taking interpersonal differences into account is critically important in order to accurately measure physiological synchronization and capture its dynamic patterns. Moreover, combining information from multiple modalities (e.g., EEG and autonomic nervous system responses) seems to be a promising approach to synchronization estimation.

Synchronization-based feedback can potentially lead to improvement of social interactions, and we envision that such feedback will be applied to many areas that could potentially benefit from it. This includes, for example, monitoring group dynamics and communication to improve collaboration efficiency among workers, monitoring teacher-student engagement to improve education, monitoring therapist-client engagement to improve mental health counseling, and much more.

## Author contributions

All authors listed have made a substantial, direct, and intellectual contribution to the work and approved it for publication.

## Funding

This work was supported in part by the National Science Foundation under grant no. 2007908.

## Conflict of interest

The authors declare that the research was conducted in the absence of any commercial or financial relationships that could be construed as a potential conflict of interest.

## Publisher's note

All claims expressed in this article are solely those of the authors and do not necessarily represent those of their affiliated organizations, or those of the publisher, the editors and the reviewers. Any product that may be evaluated in this article, or claim that may be made by its manufacturer, is not guaranteed or endorsed by the publisher.
